# Deciphering the Code between Air Pollution and Disease: The Effect of Particulate Matter on Cancer Hallmarks

**DOI:** 10.3390/ijms21010136

**Published:** 2019-12-24

**Authors:** Miguel Santibáñez-Andrade, Yolanda I. Chirino, Imelda González-Ramírez, Yesennia Sánchez-Pérez, Claudia M. García-Cuellar

**Affiliations:** 1 Subdirección de Investigación Básica, Instituto Nacional de Cancerología, San Fernando No. 22, Tlalpan, México CP 14080, DF, Mexico; msantrade@ciencias.unam.mx (M.S.-A.); igonzalez@incan.edu.mx (I.G.-R.); 2 Unidad de Biomedicina, Facultad de Estudios Superiores Iztacala, Universidad Nacional Autónoma de México, Los Reyes Iztacala, Tlalnepantla CP 54090, Estado de México, Mexico; irasemachirino@gmail.com

**Keywords:** air pollution, particulate matter, cancer hallmarks

## Abstract

Air pollution has been recognized as a global health problem, causing around 7 million deaths worldwide and representing one of the highest environmental crises that we are now facing. Close to 30% of new lung cancer cases are associated with air pollution, and the impact is more evident in major cities. In this review, we summarize and discuss the evidence regarding the effect of particulate matter (PM) and its impact in carcinogenesis, considering the “hallmarks of cancer” described by Hanahan and Weinberg in 2000 and 2011 as a guide to describing the findings that support the impact of particulate matter during the cancer continuum.

## 1. Introduction

The purpose of this review is to provide the existing elements regarding the effect of particulate matter (PM) on cancer, displaying the evidence in the context of every cancer hallmark, and describing the role and impact of PM on the carcinogenic process. To accomplish our aim, we compiled the research articles that support the carcinogenic effect of PM exposure and its role during the acquisition of cellular advantages described in each cancer hallmark proposed by Douglas Hanahan and Robert Weinberg in 2000 considering certain biological capabilities required during carcinogenesis, in a hierarchical series of conducting steps [1,2]. These capabilities include sustained proliferative signaling, evasion of growth suppression, resistance to cell death, acquisition of replicative immortality, angiogenesis induction, and activation of invasion and metastasis. The acquisition and retention of these capabilities falls mainly in a feature known as genomic instability, a driving force responsible for the genetic heterogeneity in cancer. 

## 2. Particulate Matter Generalities

PM is distributed according to its size through the respiratory tract, being coarse particles deposited in upper airways, while fine particles are deposited in deeper airways [3]. Although the characterization of PM_10_ (particles with an aerodynamic diameter equal to or less than 10 µm), PM_2.5_ (particles with an aerodynamic diameter equal to or less than 2.5 µm), or ultrafine particle (UFPs, particles with an aerodynamic diameter equal to or less than 0.1 µm) fractions provide more accuracy about the biological effect of certain components included in each group, PM_2.5_ excludes a high percentage of the inhalable fraction. The content of PM includes inorganic, organic, and biological compounds, originating from natural and anthropogenic processes [4]. Together, dust, soot, metals, salts, polycyclic aromatic hydrocarbons (PAHs), aromatic amines, endotoxins, and fungi form a complex heterogeneous mixture in PM, capable of inducing alterations in cellular homeostasis along the respiratory system and influencing the emergence of lung diseases [3,5]. 

In 2013, the International Agency for Research on Cancer (IARC) catalogued air pollution as a carcinogen for humans in Group 1 [6], and PM is one of the main components. Epidemiologic studies suggest that PM increases the incidence and mortality of different respiratory diseases, including lung cancer [7,8]. However, the specific role of PM as tumor initiator ortumor promoter has been widely debated.

### 2.1. Why Does Air Pollution Differ from Other Carcinogens?

Although air pollution (and, by addition, particulate matter) is classified in Group 1 by the IARC, the cellular mechanisms related to carcinogenesis differ from other agents included in this group. Carcinogenic agents such as virus, benzene, asbestos, benzo[a]pyrene, tamoxifen, to mention just a few, follow different routes to induce cell alterations and, thus, carcinogenesis. Air pollution is the only carcinogen in Group 1 that is not an isolated compound that can be analyzed as a pure molecule. By contrast, air pollution is a mixture of components of organic and inorganic nature mixed with aerosols, and some of the isolated components exhibit high toxicity and/or carcinogenic potential. However, the evaluation of the mixture sometimes shows null or opposite effects compared to the effects caused by those compounds tested individually. Air pollution contains some compounds classified by the IARC as carcinogens including benzo[a]pyrene and benzo[a]anthracene, as well as other genotoxic and non-genotoxic compounds. It seems that, taken together, the mechanism of carcinogenicity induced by air pollution is a mixture of multiple cellular responses, revealing synergies and antagonisms between components. Thus, testing a carcinogen with multiple components and, therefore, multiple effects, becomes more complex than other members classified in Group 1 by the IARC.

### 2.2. If Air Pollution Is Classified as a Carcinogen by the IARC and Gives Rise to Many of Cancer’s Hallmarks, What Is Missing to Understand the Mechanism of Air Pollution as a Carcinogen?

Seven years have passed since the IARC catalogued air pollution in Group I as carcinogen to humans. The decision that it be catalogued in this group is mostly defined by classic epidemiological studies that provided substantial evidence regarding the impact of particulate matter on human health, and the association with lung cancer incidence, followed by toxicological evidence that corroborated the molecular alterations necessary for lung carcinogenesis. Here, we analyzed some of the hallmarks that could partially explain the association of particulate matter exposure and lung cancer incidence. However, we identify at least four steps involved in carcinogenesis that remain indecipherable.

## 3. Evidence of PM Impacts on Different Cancer Hallmarks

### 3.1. Sustained Proliferative Signaling

Chronic proliferation is by far the main feature of cancer cells necessary to start malignant transformation. This is achieved by a strong regulation in the signaling oriented toward initiating and maintaining cell growth promotion and consecutive entry to cell-cycle phases, particularly during DNA synthesis (S phase) and mitosis (M phase), stages where the cell duplication rate is most evident. Cancer cells are capable of inducing both paracrine and autocrine induction of growth factor signaling as well as downstream pathways of cell proliferation, leading to a constant cell duplication and, thus, promoting changes in tissue architecture and function. Moreover, signaling pathways involved in cell proliferation are usually over induced, from growth factors, receptor tyrosine kinases, associated small GTPases, downstream kinases, transcription factors, and cell cycle effectors.

PM_2.5_ exposure has been associated with sustained proliferation. Pulmonary cell lines widely used in the study of the effects of air pollution such as A549, H1299, and BEAS-2B cells exhibit larger populations after treatment with PM [9]. An increase in the percentage of cells in S-phase distribution has been reported in nasal epithelial cells from individuals exposed to air pollution, as well as in pulmonary cells treated in vitro with PAHs present in PM_10_ and PM_2.5_ [10,11,12,13], exhibiting an elevated frequency of 5’-bromodeoxyuridine incorporation (unscheduled DNA synthesis), among other recognized cell cycle controllers and signaling pathways [14] that lead to cell proliferation after PM exposure [15,16] or some of PM components such as benzo[a]pyrene (BaP) [17,18]. In addition, PM_2.5_ exposure also induces the cell proliferation of lung epithelial cells through the release of exosomes, having an impact on lung tumor development [19].

Other PM_2.5_ components such as PAHs and metals can be internalized by cells not only in the cytoplasm but also in the mitochondria and nuclei, and this uptake is related to the activation of cell signaling pathways to promote the associated cell proliferation [20,21].

### 3.2. Cell Death Resistance

PM_2.5_ treatment prevents mitochondria-driven apoptosis in cell lines and primary cultures of human bronchial epithelial cells, exhibiting reductions in the frequency of morphologic changes associated with apoptosis such as cell size decrease, chromatin condensation, and formation of apoptotic bodies, an effect which is associated with PAHs [22]. Moreover, surviving cells after BaP exposure have the ability to form tumors in nude mice, indicating a role of the anti-apoptotic phenotype in cell transformation [23]. The combined effect of BaP and air pollution gases increases the levels of anti-apoptosis proteins and decreases the levels of pro-apoptotic proteins in the lung fibroblast cell line MRC-5 [24]. PM_2.5_ exposure also produces an anti-apoptotic effect resulting from both PAHs and transition metals, in an additive fashion. In fact, the anti-apoptotic effect of metals was predominant in metals and enhanced by certain PAHs. Iron in PM_2.5_ is capable of activating NRF2, followed by a repression of genes involved in cell death pathways [25,26]. Anti-apoptotic signaling has also been attributed to PAHs present in PM_10_ and PM_2.5_, followed by the production of ROS by PAH metabolism. It appears that PM exposure represents a strong factor that dictates cell fate, with the participation of ROS, PAHs and metals in cell death induction [27].

PM_2.5_ also causes an evasion of apoptosis in BEAS-2B cells through the PI3K-AKT pathway [20]. Interestingly, UFPs exhibit low levels of apoptosis, suggesting that the particles may cause impairment of mitochondrial–nuclear crosstalk, causing mitochondrial membrane depolarization, altered mitochondrial respiratory chain enzyme activity, and a reduction in the mitochondrial DNA copy number, altering redox homeostasis and inducing cell survival [28]. Exposure to PM_10_ and PM_2.5_ activates the NF-κB promoter and increases the levels and phosphorylation of NF-κB and its DNA binding activity, causing an anti-apoptotic effect on A549, BEAS-2B, murine alveolar macrophage RAW 264.7, and human umbilical vein endothelial cells (HUVEC) [26,29,30,31,32,33,34,35]. Experiments using organ-level lung microfluidic systems have reported the induction of the anti-apoptotic pathways in lung epithelial cells [20]. The anti-apoptotic effect induced by PM_10_ and PM_2.5_ has been deeply investigated, and several signaling pathways including those involving oxidative stress are described [26,33,34,35,36,37]. 

Autophagy is another cell-physiological response involved in apoptosis resistance. PM_2.5_ induces this process by the inhibition of the PI3K-AKT-mTOR signaling pathway in BEAS-2B cells [38], and the inhibition of autophagy induces apoptosis and cytotoxicity in A549 cells treated with PM_2.5_ [39]. In addition, other mechanisms have been implicated in the autophagy induced by PM exposure in lung cell lines including long non-coding RNAs [40,41].

### 3.3. Angiogenesis Induction

PM_10_ induce the formation of multiple microvessels and a dense inflammatory infiltrate when tested in the chick embryo chorioallantoic membrane assay [42]. BaP contained in PM causes early upregulation of both AKT kinase and HIF-1α, in A549 cells and lung fibroblasts [17]. Fine PM induced endothelial cell barrier disruption measured by trans-endothelial electrical resistance. PM_2.5_ induces endothelial cell cytoskeleton rearrangement causing a disruption of vascular integrity [43]. PAHs and metal components in PM_2.5_ promote the activation of pro-angiogenic factors [20,21]. Mice exposed to PM showed an increased number of tumor nodules and elevated expressions of VEGF. Measurement of angiogenesis from blood serum using an angiogenesis antibody array revealed increased levels of 12 angiogenesis factors in mice after PM_2.5_ treatment [44]. Exposure to PM_2.5_ has an impact on the methylome of primary human bronchial epithelial BEAS-2B, which impacts the expression of genes functionally linked to angiogenesis, indicating a prominent role in lung cancer signaling pathways [45].

### 3.4. Activation of Invasion and Metastasis

The regulatory program that orchestrates invasion and metastasis in cancer has been defined as the epithelial–mesenchymal transition, and the PM_2.5_-deregulated expression of genes caused by PAHs found in PM_2.5_ includes cancer progression programs [46]. In addition, PM_2.5_ exposure activates downstream inducers of epithelial–mesenchymal transition in lung cancer cells [47]. Chronic PM exposure further induced notable epithelial mesenchymal transition morphology [48]. Studies on chronic exposure in animal models have also reported the carcinogenic mechanisms of PM_2.5_ in the induction of epithelial–mesenchymal transition, but PM_2.5_ organic extract also induces different degrees of epithelial–mesenchymal transition progression and increased invasion ability in BEAS-2B cells in a concentration-dependent manner [49]. On the other hand, PM_2.5_ exposure promotes neutrophilic inflammation and tumor lung metastasis in mice [50], but PAHs, present in both PM_10_ and PM_2.5_, also promote lung cancer cell metastasis [51] and, specifically, PM_2.5_ treatment promotes atypical hyperplasia of bronchiolar epithelium in vivo [52]. Lung metastasis related to air pollution exposure has been associated with methylation of the epithelial–mesenchymal transition genes [53].

Multiple assays have demonstrated the effect of PM on migration and invasion pathways in cancer cells [54]. Data from specific toxicological in vitro assays and global transcriptome profiling show that cells exposed to PM_10_ and PM_2.5_ exhibit an increase in the activity of proteases, as well as an invasive phenotype, associated with the upregulation in metalloproteases [9,55,56]. Experiments where transepithelial electrical resistance is measured show that PM components increase cell permeability. Metalloprotease deregulation and other signaling pathways have led to enhanced ability of invasion and orthotopic lung carcinoma metastasis in mice [57]. Thus, PM_10_ and PM_2.5_ are a potent signal transducer, deregulator of non-coding RNAs, and activator of the transcription of key regulators of the epithelial–mesenchymal transition, which are strongly linked to tumor progression and metastasis [35].

### 3.5. Oxidative Stress and Inflammation

A common condition observed in solid tumors is the infiltration of immune cells with the subsequent induction of oxidative stress that contributes to the promotion of multiple cancer hallmarks. Regardless to the particle size, PM induces the production of ROS in acellular conditions, cultured cells, or animals. Consequently, in cellular and in vivo models, PM exposure leads to oxidative damage in lipids, in proteins, and, importantly, in DNA, which in turn initiates inflammatory responses [58]. However, PM exposure has an inflammogenic potential that triggers oxidative stress. For instance, PM_2.5_ induces the activation of NADPH oxidase in lung epithelial cells [59] and in the middle cerebral artery of rats [60]. This enzyme produces superoxide anion that is a free radical and precursor of hydrogen peroxide, making a clear contribution to ROS production. Additionally, PM also induces an ROS increase derived from mitochondria [61], and it has been well demonstrated that mitochondrial ROS production contributes to oncogenic transformation, promoting tumorigenesis [62]. For instance, induction of ROS by PM_10_ and PM_2.5_ is associated with disruption of cytoskeletal components, production of proinflammatory cytokines, and promotion of a concentration- and time-dependent release of TNF-α and IL6 [63,64]. Transition metals contained in PM_10_ and PM_2.5_ are partially responsible for TNF-α and IL6 release in macrophages [65]. However, other components of PM are responsible for inflammatory responses, including PAHs [26], activation of AHR [46], upregulation of CYP1A1 and CYP1B1 genes [66], hypomethylation in genes linked to cytokine and immune responses [45], upregulation of miR-146a [67], and suppression of a long non-coding RNA (lnc-PCK1-2:1) [68]. The inflammatory response induced by PM or by some PM components has been well demonstrated in several cell linages including blood mononuclear cells [69], natural killer cells [70], T cells [71], human pulmonary fibroblasts [72], but also in lung tissue of rats exposed to PM_10_ [73] and circulating monocytes and T lymphocytes from humans environmentally exposed [74], as well as in BALB/c mice [75].

The above information provides clues about the potential contribution of oxidative stress and inflammation induced by PM as a contributor to cancer promotion. Nevertheless, some exceptions of inflammatory responses have been reported such as the study of Farina et al., in which PM_2.5_ collected in Milan during winter 2008 failed to induce the massive inflammation commonly seen in mice exposed to three instillations in six days [75].

### 3.6. Genome Instability

PM_10_ and PM_2.5_ increase the frequency of sister chromatid exchanges in individuals exposed to sources of air pollution [76]. An increase in the levels of DNA damage marker 8-hydroxy-2′-deoxyguanosine, tail DNA, and micronucleus frequency has been reported after occupational exposure to PM_2.5_, suggesting a negative effect of long-term exposure to high air pollution on DNA integrity [77]. However, DNA damage induced by oxidative stress, deregulation of DNA repair enzymes, methylation of genes for maintaining chromosome structure, and deregulation of post-translational histone modifications associated with PM exposure or its components has been proposed to be linked to genome instability [78]. PM_10_ exposure induces changes in miRNA expression profiling in A549 cells. From those significantly changed, mir-324-3p and mir-6743-3p were upregulated, having a negative impact on genes involved in both DNA damage recognition and DNA damage repair pathways [79]. Methylome and transcriptomic analysis reveals that PM_2.5_ induced alterations in the DNA methylation pattern, including differentially methylated CpGs in the promoter region associated with CpG islands. Analysis of DNA methylation alterations at genome as well as gene levels has been performed in lung cancer tissues of patients from regions with severe air pollution, providing evidence of global hypomethylation, accompanied by a reduction in the expression of DNA methyltransferases DNMT3A and DNMT3B. These results suggest an important role of air pollution in the methylome, emphasizing that DNA methylation alterations caused by PAHs present in PM may be important factors for genomic instability.

**Figure 1:**
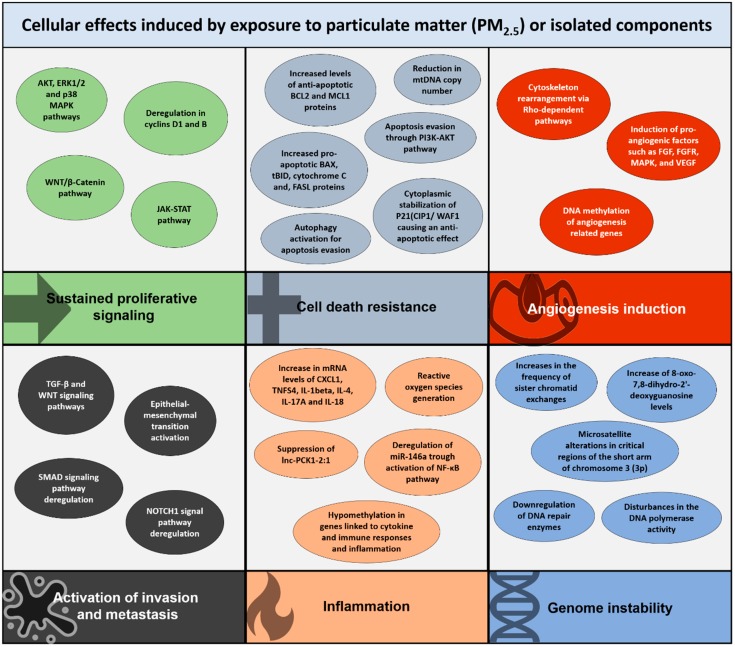
Particulate matter (PM) with an aerodynamic diameter less than 2.5 micrometers (PM_2.5_) or some of its components, such as polycyclic aromatic hydrocarbons (PAHs), have an impact on cell signaling pathways or cellular deregulations considered as key events during tumor development and known as hallmarks of cancer. However, some of these hallmarks are still negative for PM_2.5_ exposure, including induction of mutations, replicative immortality acquisition, and immune cell destruction.

Together, this section provides some of the possible cellular mechanisms by which PM exposure could promote the hallmarks of cancer. Figure 1 summarizes key events induced specifically by PM_2.5_ exposure commonly seen during cancer development. 

## 4. Toward a Comprehensive Understanding of the Effect of PM during the Cancer Continuum

The role of PM in cancer development has been studied in different contexts, giving us valuable information in order to determine the impact of air pollution on human health and, therefore, giving us the opportunity to take actions against an environmental factor catalogued as definitive carcinogen. PM has an active participation in several processes routed to the development of human tumors, promoting the acquisition of certain biological capabilities required during the cancer continuum. However, emerging approaches to study the effect of particulate matter in vivo and in vitro have increased the complexity in the understanding of its toxicological effects. While pioneer studies established essential evidence regarding the effect of PM on specific cellular processes or cancer pathways, new approaches focused on the study of global genomic profiling displays the complexity behind the gene–environment–disease interaction. In addition, we cannot dismiss that ethical issues related to animal experimentation has led the scientific community to develop alternative testing models including 2D and 3D cell cultures and organ-on-chip technology [80]. However, some proven effects such as congenital alterations induced by PM exposure [81] or by in silico or in vitro approaches maybe set aside.

Below, we discuss some aspects we considered important for moving forward to a better understanding of the role of particulate matter in cancer. 

### 4.1. Absence of Mutational Fingerprints on DNA and Growth Suppression

One of the most incomprehensible steps in the carcinogenesis associated with particulate matter exposure is the absence of mutational fingerprints on DNA. Indeed, particulate matter activates pathways oriented to “protecting” the integrity of cellular processes, such as activation of P53, RB, and other tumor suppressor genes. Many of these genes have been described in cancer as “gatekeepers”, meaning that they are the main barrier in charge of maintaining the stability of the genome. Loss of function of these genes has been associated mainly with mutations in their sequence. In fact, certain regions have been characterized as “hotspots”, because they are highly mutated in different types of cancer. Currently, there are not mutations described in these genes as a product of particulate matter exposure, and perhaps the experimental design is unable to completely simulate the chronic exposure.

### 4.2. Replicative Immortality, Cellular Energetics, and Immune Cell Destruction

Although there are some epidemiological works that report an effect of PM on the leucocyte telomere length of individuals, the results show a decrease in this biomarker, suggesting a negative effect of particulate matter on cell viability. Telomere shortening implies cellular senescence, and this phenotype activates cell death pathways in order to avoid alterations such as dicentric chromosomes or DNA double-strand breaks. Thus, evidence points toward an activation of control mechanisms directed to maintaining chromosome stability.

On the other hand, direct examples of evidence regarding the effects of PM on promoting cellular metabolism alterations commonly seen in cancer are limited, but recently a deregulation of 44 proteins related to energy metabolism and mitochondrial activity were reported in brain of rats exposed to different sizes of PM (higher aerodynamic size than 0.1 micrometers, between 2.5 and 10 micrometers, and less than 0.15 micrometers) for one month [82], and deregulation in Krebs cycle, glucose, and lipid metabolism were detected in liver of mice exposed to PM_2.5_ for 16 weeks [83]. 

The question of the evasion of immune cell destruction as an effect of PM also remains unexplored. The immune system plays an important role in detecting and eradicating the formation and growth of cancer cells in different stages, from initial formation to advanced-stage tumors and metastases. Solid tumors, such as lung cancer, have the ability of avoid detection by the immune system. Because particulate matter plays an important role in the inflammatory response, this hallmark should be considered in future approaches.

**Figure 2:**
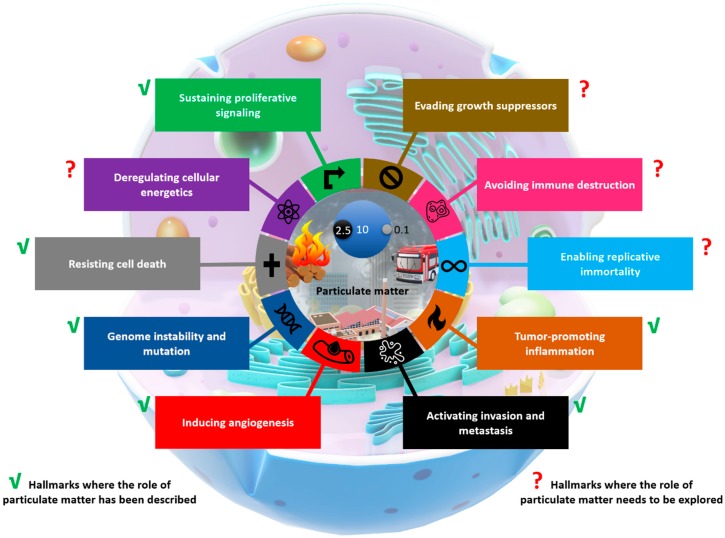
Particulate matter and its role in cancer hallmarks. Representation of the role of particulate matter in the induction of the cancer hallmarks proposed by Hanahan and Weinberg in 2000 [1] and 2011 [2].

Figure 2 summarizes the evidence regarding the effect of particulate matter on cancer hallmarks. As discussed previously, the scientific literature supports the idea that particulate matter plays an important role in establishing certain phenotypes required for tumor initiation, promotion, and progression. On the other hand, the lack of evidence in certain phenotypes highlights the areas of opportunity and the necessity for further studies to clarify the impact of air pollution during tumorigenesis. 

### 4.3. Research to Be Conducted in the Immediate Future

#### 4.3.1. Chronic Models 

The effects of air pollution are related to chronicity of exposure. A more suitable context to study the effect of particulate matter on cancer development is the experimental design with animal models. Particulate matter exposure in animals offers several research advantages, in terms of variable control and follow-up during the timeline. In fact, in vivo studies could provide evidence regarding both the cellular and individual impact of particulate matter, following all cancer hallmarks and their appearance during the multistep process of the disease. However, this approach has been considered the least, perhaps because follow-up implies the inclusion of a considerable number of individuals, submitted to long exposure and maintenance periods and therefore more extensive in terms of publishing, making research in animals a less cost-effective strategy. In addition, a more realistic exposure must include air chambers of exposure instead of instillation models. In terms of in vitro models, sustained exposures under non-cytotoxic conditions using, for instance, air–liquid interface systems seem more suitable than submerged cell cultures. We argue that exposure to low concentrations of particulate matter is needed because human exposure occurs in the absence of immediate intoxication symptoms. However, one disadvantage of in vitro models is the use of transformed cell lines that normally have some mutations. Unfortunately, primary cell cultures are not suitable for chronic exposures because the propagation of a cell population by subculturing has a limited replicative life span.

#### 4.3.2. Air Pollution as a Mutagenic Agent Needs to Be Addressed Using Innovative Technology and Epigenetic Alterations

Air pollution exposure has well-demonstrated genotoxic effects; however, it is a carcinogen without mutagenic fingerprints not yet demonstrated. Although some PAHs found in particulate matter have been characterized as mutagenic/carcinogenic, it appears that the effect is masked in mixture. There is only one study’s evidence associated with the mutagenic potential of particulate matter, obtained using the Ames test, which is a fast biological assay used to estimate the mutagenic/carcinogenic effect of a compound when compared with animal models. However, the major limitation of that test is the model used, since it is performed in prokaryote cells and which is perhaps not the best way to demonstrate mutations in eukaryote cells. Moreover, the fact that particulate matter does not cause mutations directly does not necessarily mean that PM is unable to induce them. Perhaps the technology was unable to detect them, or more precise techniques such as sequencing were less accessible some years ago. Today, however, a spectrum of more powerful tools is available, and at lower costs. In addition, the role of epigenetic alterations and mutations has not been completely revealed, specifically in the field of air pollution effects. However, recently, epigenetic alterations were found in epidemiological studies related to air pollution exposure, and some PM components such as PAH and nitric dioxide deregulated methylation patterns in genes related to breast cancer development [84]. 

### 4.4. Beyond the Epidemiological Studies, Other Risk Factors May Be Missing in the Research Conducted to Reveal the Mechanism of Air Pollution Carcinogenicity 

There is an experimental gap in the study of air pollution, and mainly particulate matter, as a carcinogenic factor. Although the IARC classification of carcinogens in humans is based on the evidence provided by epidemiological studies, as well as in animal and cellular models, epidemiological results are sometimes the most important in terms of actions. However, many associations found in epidemiological studies do not always correlate with findings in animal models or in vitro studies, perhaps because some key factors are also relevant for triggering the association of particulate matter and cancer development. These key factors are missing in the in vivo, in vitro, and in silico studies. 

This situation creates certain uncertainty, since both approaches have pros and cons. For example, although cohort studies provide strong evidence, studies in humans always include confusing factors that researchers are unable to control. Risk factors associated with lung cancer, such as smoking, are considered and normalized in a study population, but many other sources or expositions are sometimes unknown. Furthermore, epidemiological studies operate on a scale that cannot provide information relevant to understanding the acquisition of hallmarks during cancer development. On the other hand, in vitro studies analyze cellular responses and provide further evidence about the biological impact of particulate matter. In this case, the toxicological studies operate on a scale capable of providing information about the acquisition of certain cancer hallmarks. However, toxicological studies tend to isolate the effect of the risk factor studies, and therefore provide punctual evidence. We can summarize the disadvantages and challenges of both models in one sentence: We need more efforts to translate the impact of particulate matter described in humans to the bench, in order to provide clues focused on understanding its carcinogenic potential during tumorigenesis. It is possible that epidemiological studies may soon disclose key risk factors that, together with air pollution exposure, lead to carcinogenic effects. 

### 4.5. Air Pollution Exposure Is Not Considered as a Risk Factor for Failure in Lung Cancer Treatment 

Air pollution exposure is a risk factor for lung cancer development; however, the conventional therapy never considers all the molecular, cellular, and biochemical alterations already induced by particulate matter. Cancer treatment schemes such as radiotherapy and chemotherapy are based on the principle of causing “cytotoxicity” by the generation of genome instability. The action mechanism of radiotherapy is based on causing a higher rate in double-strand breaks in DNA, accompanied by ROS generation, causing DNA damage and, thus, cell death. On the other hand, the action mechanisms of chemotherapy agents such as cisplatin and paclitaxel are the generation of DNA damage by the formation of DNA adducts or the generation of chromosomal instability. Because particulate matter is capable of inducing DNA damage in the same way that therapy does, it is important to determine how chronic exposure to an environmental risk factor that induces genomic instability, and cancer, is capable of generating resistance to certain cancer treatments, where the threshold for cytotoxicity induction is modified, giving a cancer-cell population the capacity to tolerate higher doses of treatment and causing less response to treatment in cancer patients. 

## 5. Final Remarks

In summary, exposure to particulate matter induces multiple hallmarks of cancer seen during tumor development with the exception of the absence of mutational fingerprints on DNA, the acquisition of replicative immortality, and the avoidance of immune destruction not only for lung cancer development.

These are the key gaps that must be addressed to understand the mechanism of air pollution as a carcinogen. Research that may help to reveal those mechanisms is the use of chronic models and animal and in vitro studies, as well as in silico approaches together with epidemiological evidence, which would help to understand the role of particulate matter during the cancer continuum. 

We also identified the conclusion that air pollution is a risk factor for lung tumorigenesis. However, during lung cancer therapy, we still cannot translate the findings into a more personalized treatment for patients highly exposed to particulate matter.

## 6. Methods

This review was conducted by searching existing published studies in English using three databases, namely PubMed, Web of Science, and Scientific Electronic Library Online (SciELO). Terms used during the searches considered the combination (Boolean operator “AND”) of three categories of concepts. The first category corresponded to terms such as “air pollution”, “particulate matter”, “PM_10_”, and “PM_2.5_”. The second category corresponded to terms such as “oxidative stress”, “proliferation”, “tumor suppression”, “cell death”, “replicative immortality”, “angiogenesis”, “invasion”, “metastasis”, “inflammation”, and “genomic instability”. The third category included terms such as “cancer”, “carcinogenesis” “transformation”, and “tumors”. After searching, our inclusion criteria were based on the following parameters. We included epidemiological, in vivo, and in vitro research studies involving particulate matter (PM_10_, PM_2.5_, or UFP) and its impact on pathways associated with cancer hallmarks. Finally, studies considered in this review included those publications supporting the role of particulate matter in cancer hallmarks. Research publications whose results did not correspond to the topic of discussion were discarded for this review manuscript.
